# Optimization of Micro and Nano Palm Oil Fuel Ash to Determine the Carbonation Resistance of the Concrete in Accelerated Condition

**DOI:** 10.3390/ma12010130

**Published:** 2019-01-03

**Authors:** Wei Le Tang, Han-Seung Lee, Vanissorn Vimonsatit, Trevor Htut, Jitendra Kumar Singh, Wan Nur Firdaus Wan Hassan, Mohamed A. Ismail, Asiful H. Seikh, Nabeel Alharthi

**Affiliations:** 1 School of Civil and Mechanical Engineering, Curtin University, Bentley, Perth 6102, Australia; weile.tang@postgrad.curtin.edu.au (W.L.T.); V.Vimonsatit@curtin.edu.au (V.V.); trevor.htut@curtin.edu.au (T.H.); 2 Department of Architectural Engineering, Hanyang University, 1271 Sa3-dong, Sangrok-gu, Ansan 15588, Korea; ercleehs@hanyang.ac.kr; 3 Department of Civil and Construction Engineering, Faculty of Engineering and Science, Curtin University Malaysia, Miri 98000, Sarawak, Malaysia; wannurfirdaus@postgrad.curtin.edu.my (W.N.F.W.H); mohamed.ismail@vip.henu.edu.cn (M.A.I.); 4 Civil Engineering Department, Miami College of Henan University, Jinming Avenue No. 1, Kaifeng 475004, Henan, China; 5 Centre of Excellence for Research in Engineering Materials, King Saud University, P.O. Box 800, Riyadh 11421, Saudi Arabia; alharthy@ksu.edu.sa; 6 Mechanical Engineering Department, King Saud University, P.O. Box 800, Riyadh 11421, Saudi Arabia

**Keywords:** carbonation depth, concrete, microstructure, morphology, palm oil fuel ash, sorptivity

## Abstract

The carbonation rate of reinforced concrete is influenced by three parameters, namely temperature, relative humidity, and concentration of carbon dioxide (CO_2_) in the surroundings. As knowledge of the service lifespan of reinforced concrete is crucial in terms of corrosion, the carbonation process is important to study, and high-performance durable reinforced concretes can be produced to prolong the effects of corrosion. To examine carbonation resistance, accelerated carbonation testing was conducted in accordance with the standards of BS 1881-210:2013. In this study, 10–30% of micro palm oil fuel ash (mPOFA) and 0.5–1.5% of nano-POFA (nPOFA) were incorporated into concrete mixtures to determine the optimum amount for achieving the highest carbonation resistance after 28 days water curing and accelerated CO_2_ conditions up to 70 days of exposure. The effect of carbonation on concrete specimens with the inclusion of mPOFA and nPOFA was investigated. The carbonation depth was identified by phenolphthalein solution. The highest carbonation resistance of concrete was found after the inclusion of 10% mPOFA and 0.5% nPOFA, while the lowest carbonation resistance was found after the inclusion of 30% mPOFA and 1.5% nPOFA.

## 1. Introduction

Durability of concrete is a major concern when exposed to aggressive environments, especially chloride and carbon dioxide (CO_2_) causing chlorination and carbonation, respectively. These ions induce the corrosion of embedded steel rebars [1]. Once the steel rebar starts to corrode, the corrosion products induce internal expansion, resulting in cracks and spalling, which leads to the failure of concrete structures [2].

Chlorination can also affect the durability of concrete more often than carbonation [3]. However, the increase of urban population density and industrialization as well as more technologies have led to higher emission of carbon, which dramatically increases the concentration of CO_2_ in the atmosphere.

The theory of carbonation is a complex process. Carbonation occurs once calcium carbonate (CaCO_3_) forms [4]. Generally, the formation of CaCO_3_ occurs when calcium hydroxide (Ca(OH)_2_) encounters atmospheric CO_2_ in the presence of water [5]. Ca(OH)_2_ is an alkaline substance, which is consumed during carbonation process; thereafter the concrete becomes more acidic, causing a reduction in the pH of the pore solution and successfully breaking down all other hydrate phases [6]. Thus, the final products become a mixture of carbonates with silicate, ferrite and aluminum-hydroxide phases. In other words, the chemistry of carbonation is always the same; however, the penetration difficulties into the concrete vary in different concrete mixtures.

The carbonation process begins when CO_2_ from the atmosphere diffuses into concrete. The gaseous CO_2_ cannot directly react with the hydrates of cement paste. However, it dissolves in water (H_2_O) and forms bicarbonate (HCO_3_^−^) ions (Equation (1)) and thereafter reacts with calcium ions (Ca^2+^) of the pore water [6]. However, since the pH value inside the concrete is high, the HCO_3_^−^ will dissociate and form carbonate (CO_3_^2−^) ions (Equation (2)). Lastly, the carbonate ions will react with the Ca^2+^ ions in the pore solution and form CaCO_3_ in Equation (3) [7]. The carbonation process is described by the following chemical equations [8]:

(1) $${\mathrm{CO}}_{2\left(g\right)}+H_{2}O\to {\mathrm{HCO}}_{3}^{-}\left(\mathrm{bicarbonate}\;\mathrm{ion}\right)+H^{+}$$

(2) $${\mathrm{HCO}}_{3}^{-}+H_{2}O\to {\mathrm{CO}}_{3}^{2-}+H_{3}O^{+}$$

(3) $$H_{2}{\mathrm{CO}}_{3}+\mathrm{Ca}{\left(\mathrm{OH}\right)}_{2}\to {\mathrm{CaCO}}_{3}+2H_{2}O$$

There are three different factors affecting the carbonation process, namely the temperature, relative humidity, and concentration of CO_2_ present in the surroundings [7]. When the temperature increases, the diffusivity of CO_2_ into concrete is amplified due to the increase of molecular activity [9]. Secondly, the relative humidity acts as the major role in determining the diffusivity of CO_2_. The highest rate of carbonation happens at the relative humidity of 50–70% [10]. The carbonation process generally occurs in the presence of water. However, if it is too wet, water acts as the obstruction for penetration of CO_2_, which in turn decreases the rate of the carbonation process. Lastly, the CO_2_ concentration in the environment is certainly the main factor controlling the carbonation process. If the atmosphere has high concentration of CO_2_, this can induce the carbonation process [11]. In the diffusion process of carbonation, CO_2_ flows from higher concentration to lower concentration. Therefore, the rate of diffusivity depends on the level of concentration. However, different surface-repairing materials are becoming prominent to reduce the carbonation effect on concrete [12,13]; but there are some issues related to post-treatment regarding cost, durability, and practical application.

Blended cement has now become very popular, owing to better performance in terms of mechanical properties as well as carbonation resistance when compared with ordinary Portland cement (OPC) [14,15]. As a result, the trend of using pozzolanic materials as partial replacement of cement is expanding [16].

The three largest producers of palm oil fuel ash (POFA) are Indonesia, Malaysia, and Thailand. In 1960, palm oil cultivation was limited to 54,000 hectares in Malaysia; however, it has substantially increased to 4.85 million hectares in 2010 and 5.39 million hectares in 2014. Moreover, in Indonesia, palm oil cultivation was 6.5 million hectares in 2012. Palm oil cultivation produces large portions of biomass waste such as fronds, effluent, leaves, trunks, kernel shell, mesocarp fiber, and empty fruit branches after the harvesting of fruits, and processing and re-plantation of palm trees [16]. These huge portions of biomass are burnt at 800–1000 °C in an industry to generate electricity [16]. The end product of this burning process is known as POFA, which is normally thrown into the landfill causing an environmental impact. Moreover, when wind is blown through the landfill, it could cause health hazards, as it is toxic to breathe [17]. Due to the high production of POFA, it is a severe risk to health conditions and environmental damage. The situation will be worse as it is forecast that the generation of POFA will tend to increase in the future. Therefore, reusing POFA from landfill is good opportunity for reducing environmental and health effects and at the same time achieving better performance of concrete.

Raw POFA has zero cost and the treating process requires only a few devices such as an oven, grinder and ball mill, and thus it is not expensive. This is helping the environment, because all unused raw POFA will be thrown into landfill, causing pollution. As a result, reusing POFA can save the environment. The cost can also be reduced by using POFA instead of cement. Cement is more expensive than POFA. Therefore, replacing cement with POFA can make concrete production cost-effective in addition to reducing the environmental load.

POFA has very good potential for improving concrete’s properties due to its chemical composition, such as calcium and silicon oxides present inside [18]. Thus, POFA is proven to be the emerging waste material for improving the durability of concrete, owing to the formation of secondary calcium silicate hydrate (C-S-H) gel through a pozzolanic reaction, which is caused by silica [19]. A certain amount of micro POFA (mPOFA) enhances the durability of concrete, which is 10–30% in replacement of OPC [20,21,22,23]. POFA shows a lower strength activity index at an early stage of curing, but it exhibits improved values at a later stage [24]. It is used in the production of high-strength concrete by reducing the average size to around 10 µm with a 0–30% replacement of OPC [22,25,26]. At the age of 28 days, the highest compressive strength was found with 20% replacement of OPC by POFA [22,25]. The inclusion of ultrafine POFA reduces the early-stage compressive strength up to 7 days and it was particularly observed for a higher amount of POFA [27]. It was also seen that if mPOFA is converted into nano-POFA (nPOFA) and included together to produce concrete, the result had a positive influence in filling out the porosity of concrete and enhancing the compressive strength [28].

According to Thomas et al. [16], POFA has been proven to bring benefits to carbonation resistance by using micro sizes of POFA. There is a high possibility of using nano-size POFA to achieve higher carbonation resistance of concrete, compared to micro POFA [29]. Therefore, this study tried to use POFA as a supplementary cementitious material for concrete. Islam et al. found that using 30% mPOFA exhibited better performance, but once the amount increased, it showed detrimental effects to the concrete [18]. Furthermore, it was found that using a little amount of nPOFA can replace a huge amount of mPOFA [30]. Therefore, in the present study, the concrete mixtures containing a low amount of nPOFA along with mPOFA were prepared and their synergistic effects on carbonation resistance under accelerated conditions were measured.

In the present study, the amount of POFA has been optimized for high resistance to carbonation. This study emphasizes the assessment of the carbonation resistance properties of concrete by incorporating micro and nano-POFA. The accelerated carbonation experiment was performed according to BS 1881-2010:2013 in 4.0% (±0.5%) CO_2_ at 20 °C (±5 °C) temperature and 55% (±5%) relative humidity [31]. In this standard method, there is a tolerance value for both temperature and relative humidity of ±5 °C and ±5%, respectively. This would not have any effect on the acceleration of carbonation if the temperature is around room temperature and relative humidity is within 50–70% [7]. Under these circumstances, a maximized effect would be gained in this research.

## 2. Materials and Methods

### 2.1. Materials

#### 2.1.1. Binders

Two types of binder, known as OPC and POFA, were used in the present study. The chemical composition of binders will be discussed in the Test Results and Discussion section.

**Figure 1:**
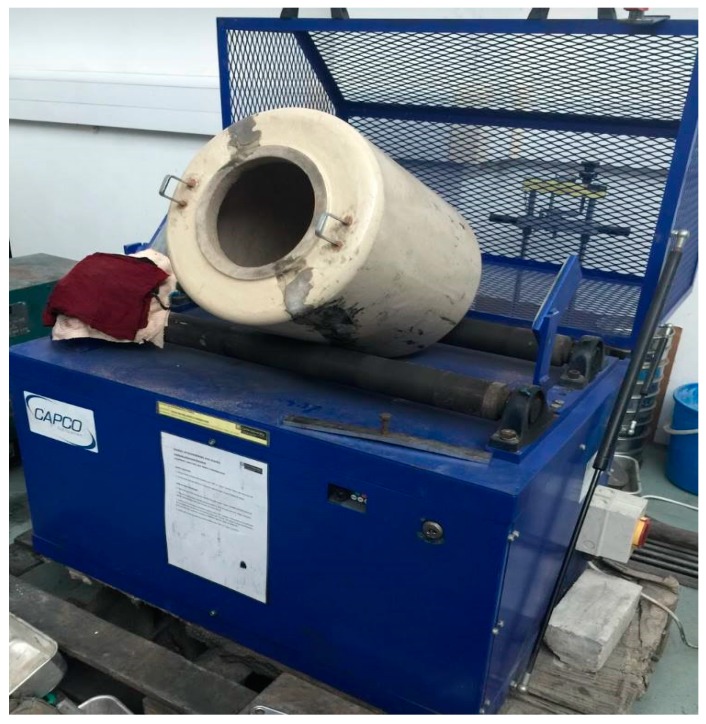
Image of high-energy ball mill.

The raw POFA was obtained from a palm oil mill at Miri, Sarawak, Malaysia, and the details about the procedure to obtain POFA are described in a recently published work [32]. The raw POFA was dried in an oven at 105 ± 5 °C for 24 h to remove the moisture. After drying, POFA was sieved through a 150 μm mesh to remove 15–22% of the coarser particles. The coarser particles include unwanted waste, such as dry branches, dry leaves, tiny pieces of palm oil shell, etc. The sieved POFA then went through the grinding process to ensure 90% passes through 45-μm size. Thereafter, the fine POFA powder was heated up to 500 °C for 1 h in a furnace. After trial and error, it was found that 500 °C was the optimum temperature which successfully removed the unburnt carbon from POFA. Several authors, such as Al-Mulali et. al. [33], Zeyad et. al. [34], and Chandara et al. [35] have also found similar results. At this stage, POFA had turned from black to brownish color [28]; this form of POFA is known as the treated mPOFA. The color had changed due to the removal of excessive carbon from POFA during the heating process. Next, the mPOFA was subjected to further grinding by using a high-energy ball mill, as shown in Figure 1, to obtain treated nPOFA [36]. The specific gravity of mPOFA and nPOFA used in this research was 2.63 and 4.65, respectively.

#### 2.1.2. High-Energy Ball Mill

A high-energy ball mill (Model 3 VS, Capco Test Equipment Company, Suffolk, UK) was used to grind the mPOFA into nPOFA [37]. A similar ball mill was used by Rizlan and Mamat [38] to produce nano-size particles. The volume of the ceramic jar was 0.032 $m^{3}$ and the total weight of the stainless steel balls was 30 kg. Three different diameters of stainless steel ball i.e., 12.7 mm, 19.0 mm and 25.4 mm with 9.0 kg, 9.0 kg and 25.4 kg weight, respectively, were used to grind the mPOFA. An mPOFA amount of 1.8 kg was loaded into the ball mill, placing it on top of the stainless steel balls and the grinding operation was performed for 5 h to obtain 1.5 kg nPOFA. The mass loss of 0.3 kg might be attributed to flying of some fine particles of nPOFA which is very light in weight.

#### 2.1.3. Aggregates

The standards used for analyzing the aggregates were ACI 211-4R [39] and ASTM C33-16 [40]. The ACI 211-4R was used to obtain the density of coarse aggregates and ASTM C33-16 was used to check the grading requirement of coarse aggregates. The coarse aggregates used in this research were crushed granite from a local source with nominal size range from 9.5 mm to 12.5 mm. The fineness modulus, specific gravity, and water absorption of the coarse aggregates were 2.3, 2.71, and 0.5%, respectively.

There were two elements used as fine aggregates, known as river sand and quarry dust. River sand was obtained from a local source of Sarawak, Malaysia. The quarry dust was incorporated into the concrete mixture due to the low fineness modulus of river sand. The fineness modulus [0.99] of river sand obtained in Miri, Sarawak, Malaysia itself did not meet the requirements in accordance with the standards; hence, quarry dust was required to be mixed with sand. The amount of river sand used was 50% and that of quarry dust was 50%. The size of the sand was smaller than 4.5 mm while the size of the quarry dust was between 4.5 mm and 9.5 mm. The fineness modulus of the combined fine aggregates (river sand plus quarry dust) was 2.67.

#### 2.1.4. Superplasticizer

A polycarboxylate ether was used as superplasticizer (SP) in this study. It met the requirements of ASTM C494-16 [41]. The selected SP was able to work for the concrete mixtures with low-cement ratio while obtaining extended slump retention.

### 2.2. Concrete Mix Proportions and Specimens Preparation

The mixture proportions of different concretes were derived based on ACI 211-4R [39]. The cement content, water/binder ratio, density of coarse aggregates, density of combined fine aggregates (sand and quarry dust), and SP dosages was 588 $\mathrm{kg}/m^{3}$, 0.35, 1093 $\mathrm{kg}/m^{3}$, 268 $\mathrm{kg}/m^{3}$ and 0.2% by weight of binder, respectively. The binder proportions and the weights of different constituent materials are shown in Table 1 and Table 2, respectively. The concrete specimens were prepared in triplicate for each mixture. The dimensions of concrete specimens used for carbonation resistance test were 280 mm × 70 mm × 70 mm. In the case of sorption test, 100 mm diameter × 50 mm height cylinders were used. These concrete specimens were cast by using prism and cylinder mold, respectively.

Mixing of the concrete was carried out by a pan mixer. The pouring of the concrete into the mold was done in three layers; compaction was done after every layer filled. The specimens were demolded after 24 h of casting and transferred to water curing at 20 ± 2 °C for 28 days.

### 2.3. Testing Procedures

#### 2.3.1. X-ray Fluorescence (XRF)

The XRF (Rigaku ZSX Primus IV, Tokyo, Japan) was used to determine the chemical compositions of OPC and POFA. For this, OPC, mPOFA and nPOFA were prepared and stored in the sealed plastic bag with 150 g of each sample.

#### 2.3.2. X-ray Diffraction (XRD)

The acquired POFA from Miri, Sarawak, Malaysia was subjected to XRD (Bruker AXS, Billerica, Germany) analysis to determine its phase composition. Prior to analysis, the samples were prepared in powder form. To collect the XRD spectrum, Cu Kα radiation (λ = 1.54059 Å) generated at 40 kV and 40 mA was used. The 2θ scan range was 20–70°. The step size was 0.015. The database of Joint Committee on Powder Diffraction Standards (JCPDS) was used to ascertain the phase composition of POFA.

#### 2.3.3. Scanning Electron Microscopy (SEM)

The determination of morphology of POFA was carried out by SEM (Philips XL 30, North Billerica, MA, USA) and operated at 15 kV. Prior to perform the SEM experiment, the specimens were mounted into the aluminum stubs. The purpose of mounting was to stabilize the sample for viewing and maneuvering in the SEM chamber. Next, the gold splutter coater was used to coat the mounted specimens. Lastly, the coated specimens were placed inside the SEM instrument. The particle size calculation was carried out by ImageJ software (version Java 1.8.0_172).

#### 2.3.4. Slump Test

The slump test was carried out according to ASTM C143 standards [42] to determine the workability of concrete mixtures.

#### 2.3.5. Carbonation Experiment: Accelerated Method

The size of concrete specimens used for the accelerated carbonation testing was 280 mm × 70 mm × 70 mm. Accelerated carbonation testing was carried out to determine the carbonation depth in accordance to BS 1881-210:2013 [31]. The concrete specimens were cast and placed inside the water curing room for 28 days. There were 10 mixtures with 3 specimens each. The total number of specimens tested was 30. Once the concrete specimens were cast and cured, paraffin wax was required to seal the top and bottom longitudinal faces and the two end faces of prism. This was to avoid multi directional penetration of CO_2_.

The accelerated carbonation experiment was performed in 4.0 ± 0.5% CO_2_, 20 ± 5 °C temperature and 55 ± 5% relative humidity in accordance with BS 1881-210:2013 [31].

Generally, the CO_2_ concentration in the atmosphere was estimated to be 0.2% while in urban or industrial areas, the CO_2_ may rise to 0.4% [43]. Since the carbonation chamber used 4.0% CO_2_, it is almost 10 times higher compared with natural process. This concluded that using 4.0% CO_2_ can accelerate the carbonation process.

**Figure 2:**
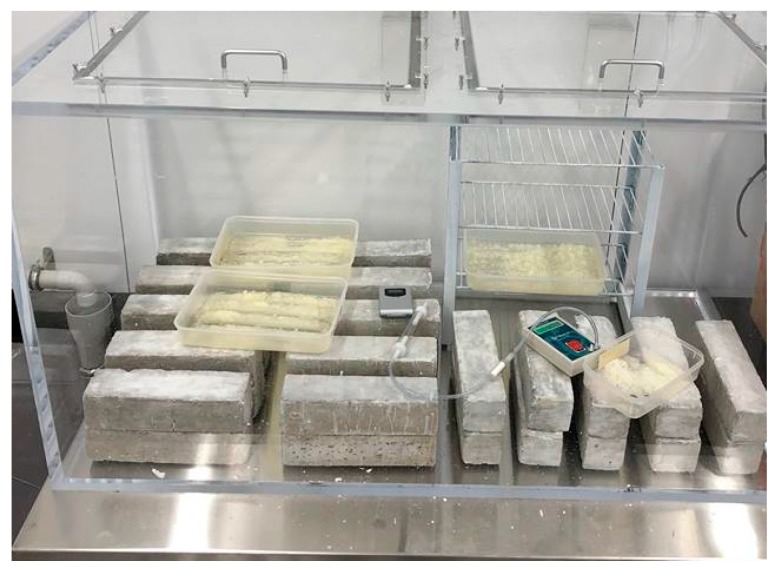
Overview of carbonation chamber.

The concrete specimens were placed inside the carbonation chamber for up to 70 days. The carbonation chamber was fabricated by Johndec Centigrade Company in Perth, Australia with acrylic box. The carbonation depth measurement was carried out after 56, 63 and 70 days. The measurement of carbonation depth before 56 days was not recorded, as the carbonation depth was too small to be seen. The overview for the set-up of carbonation chamber is shown in Figure 2.

The phenolphthalein solution was used to determine the carbonation depth of concrete. The solution was produced by using 1 g of phenolphthalein powder dissolved in a 100 mL solution of 30 mL deionized water and 70 mL of ethanol solution. After spraying on the concrete specimen, the carbonated area was colorless while the uncarbonated area changed to purple color.

#### 2.3.6. Sorptivity Test

**Figure 3:**
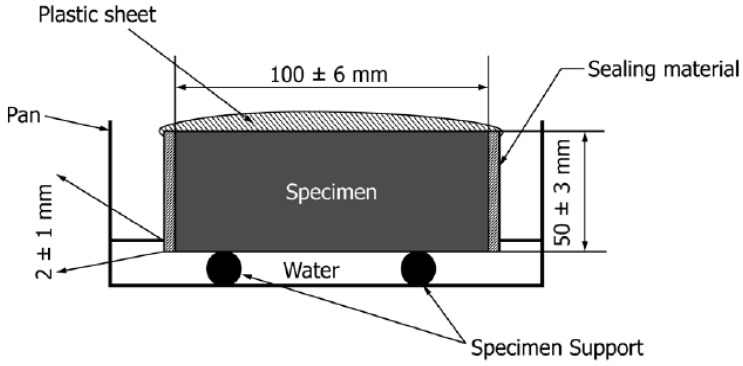
Schematic of sorptivity test procedure [44].

The concrete specimens used for sorptivity experiment were 100 mm diameter × 50 mm height cylinders. Triplicate specimens were used for each of 10 concrete mixtures; therefore, a total of 30 specimens were tested. The sorptivity test was carried out in accordance with the ASTM C1585-13 standards [44]. This test method is to determine the rate of absorption of water. Specimens were placed inside the water curing room for 28 days. After the curing process, the side surface of the specimens was sealed with plastic sheet. The test set-up is shown in Figure 3. The purpose of this testing was to measure the water absorptivity of concrete. This can show the penetrability of concrete to relate it to the penetration of CO_2_. The sorptivity measurement of the specimens with different time intervals and tolerance is shown in Table 3.

## 3. Test Results and Discussion

### 3.1. Characterization of POFA

#### 3.1.1. X-ray Fluorescence

The chemical compositions of OPC and POFA are presented in Table 4. The major chemical components of OPC are CaO and SiO_2_ while POFA contains SiO_2_ along with high SiO_2_ + Al_2_O_3_ + Fe_2_O_3_. The presence of high content of SiO_2_ in POFA indicates that it is a pozzolanic material. Table 4 also shows the loss on ignition (LOI) for both OPC and POFA. The high LOI in raw POFA is attributed to the presence of organic component but once it was treated, the amount of organic material decreased dramatically. Hence, the LOI values are significantly lower for mPOFA and nPOFA. From Table 4, it can be seen that the LOI is decreased from 10.5% for raw POFA to 1.71% and 1.60% for mPOFA and nPOFA, respectively. The lower LOI values emphasize that the quality of treated POFA improves compared with raw POFA. After the treatment of raw POFA, the LOI is reduced by 28.75% and 33.33% for mPOFA and nPOFA, respectively. Such quality improvement of POFA may increase its potential to enhance the properties of concrete.

#### 3.1.2. X-Ray Diffraction

**Figure 4:**
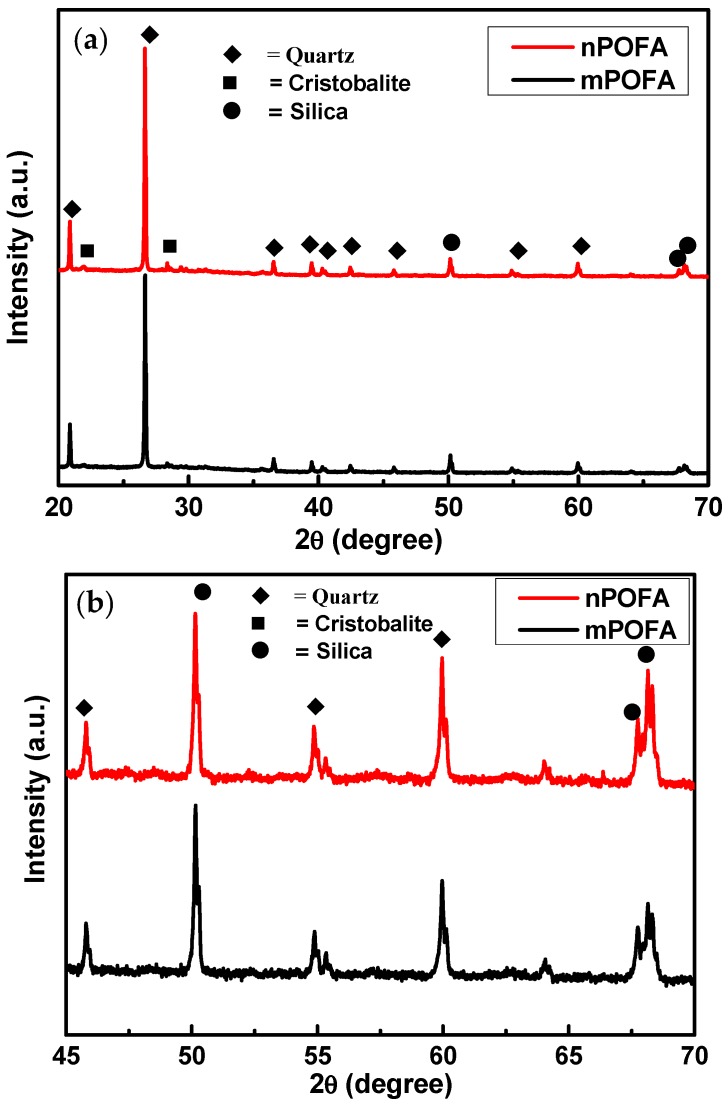
XRD of micro- and nano-POFA at 2θ = 20–70° (**a**) and 2θ = 45–70° (**b**).

The XRD of mPOFA and nPOFA are presented in Figure 4. Figure 4a shows the full range of scanning from 2θ = 20–70° where small peaks were suppressed due to high intensity of Quartz. Therefore, to make it clear, it has been plotted using 2θ = 45–70° in Figure 4b. There are some broadening in peaks and appearance of hump which suggest that POFA has certain amorphous phases (Figure 4b). From Figure 4, it can be seen that mPOFA and nPOFA contain Quartz (JCPDS = 88-2487), Cristobalite (JCPDS = 82-1410), and amorphous silica (JCPDS = 89-1665) which agree with other researchers’ work [24,28,32,45,46,47]. As the temperature was increased while treating raw POFA, the amorphous silica transformed into the crystalline phase. However, 500 °C temperature with 1 h of heating time was not sufficient to transform all amorphous silica into crystalline phase in the present study. Therefore, amorphous silica phase is observed at 2θ = 50–70°. There is a chance that this amorphous phase of silica might react with Ca(OH)_2_ liberated from cement hydration in concrete to form secondary C-S-H.

The crystallite size (*L*) of different mineral phases was calculated by the Scherrer equation as shown below.

(4) $$L=\frac{K\lambda }{\beta \times \cos\theta }$$

where λ is the X-ray wavelength in nanometer (nm), β is the peak width of the diffraction peak profile at maximum height from small crystallite size in radians and K is a constant related to crystallite shape taken as 0.9.

The crystallite size of Quartz was 68.75 nm in nPOFA whereas it was 79.1 nm in mPOFA. The crystallite size of Cristobalite was 91.7 nm and 93.8 nm in nPOFA and mPOFA, respectively. On the other hand, the crystallite size of amorphous silica was found to be 77.10 nm in nPOFA whereas it was 80.9 nm in mPOFA. These results suggest that the crystallite/grain size of nPOFA is lower than that of mPOFA.

#### 3.1.3. Scanning Electron Microscope

**Figure 5:**
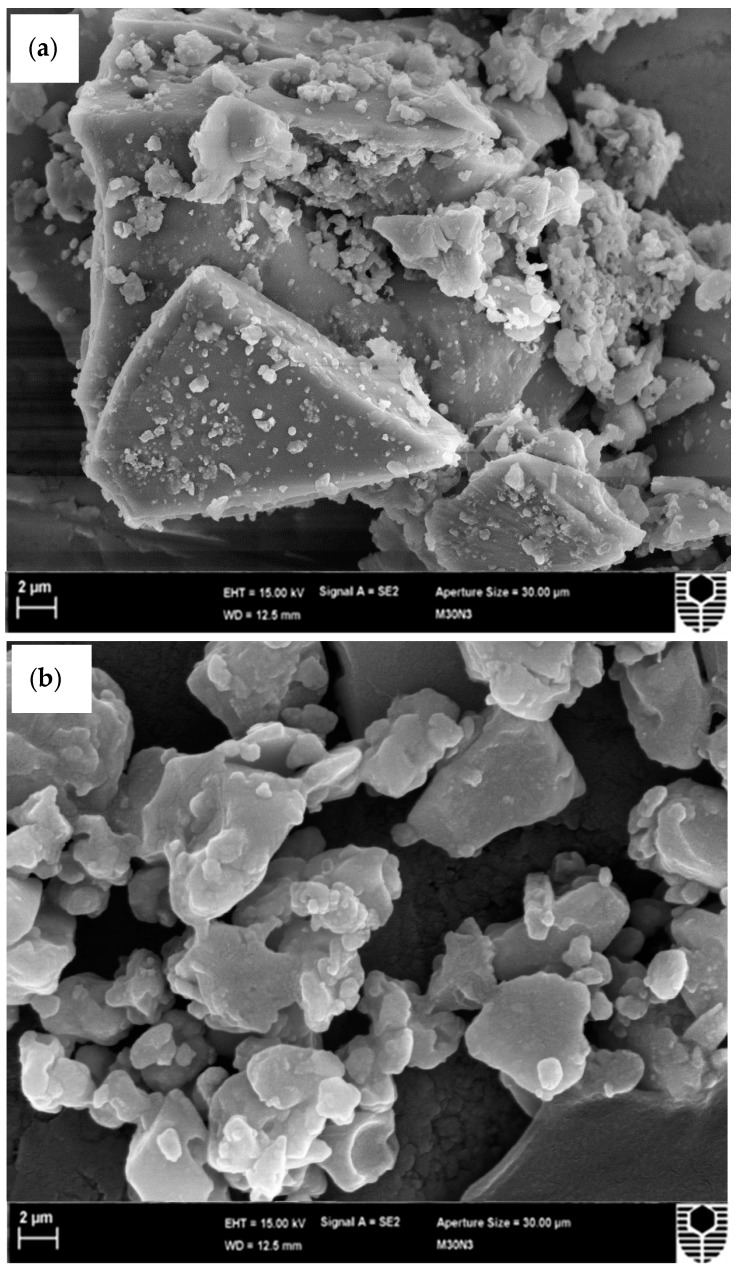
Surface morphology of micro (**a**) and nano (**b**) POFA.

**Figure 6:**
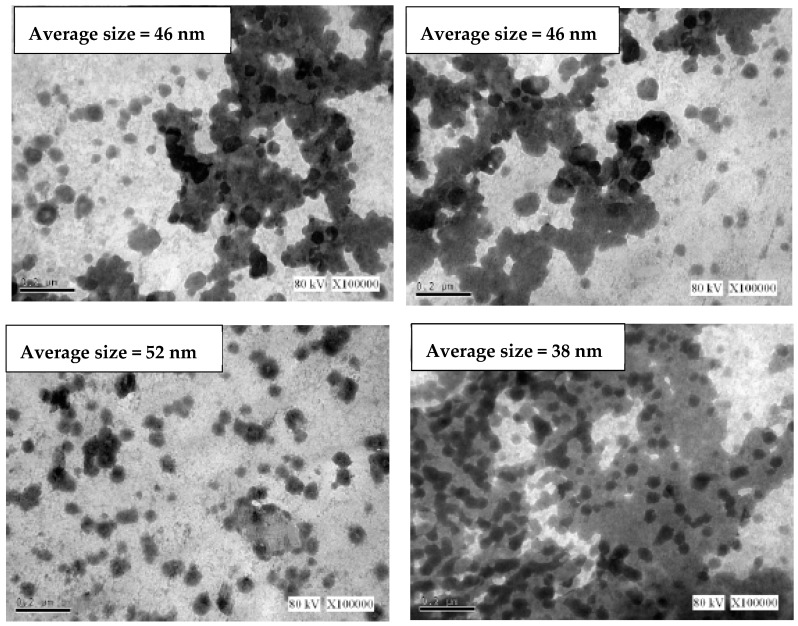
TEM image of nPOFA with average particle size at different locations [28].

The surface morphology of mPOFA and nPOFA particles are shown in Figure 5. Figure 5a shows that the mPOFA had crushed or irregularly shaped particles and found to be bigger in size compared with nPOFA. Figure 5b demonstrates that the particles of nPOFA were finer and more crushed. The nano particle size is in between 33.50 nm and 58.10 nm. The advantage of the smaller and irregular particle size is that it can improve the properties of the concrete due to the greater filling ability. However, to confirm the particle size of nPOFA, it was also characterized by transmission electron microscopy (TEM, JEOL-1230, Tokyo, Japan) and the results are shown in Figure 6. This figure also shows that the average particle size of nPOFA is around 38–52 nm at different locations [28]. The particle sizes of raw POFA and mPOFA are greater and beyond the limit of TEM. Therefore, it was not possible to perform this experiment for them.

### 3.2. Workability of Concretes

The workability of different concrete mixtures in terms of slump measurement is shown in Table 5 along with their slump loss value. The loss of slump was calculated with respect to OPC concrete (M0). From Table 5, it is evident that using both mPOFA and nPOFA increased the workability of concretes, especially M10N1 (slump loss = 15 mm), M10N2 (slump loss = 0 mm) and M10N3 (slump loss = −5 mm). The slump loss value in most of the mixtures was higher while M10N3 showed no slump loss. M10N1 showed only 10.7% slump loss, which means the workability of it was almost identical with that of OPC concrete. It was reported that the fine particles of treated POFA are adsorbed on the oppositely charged surface of cement particles which then prevent them from flocculation [48]. As a result, this would cause the cement particles to be more dispersed and, therefore, would not trap a large amount of water. In the present study, M10N2 and M10N3 contained higher amount of nPOFA which is smaller in particle size compared to mPOFA and OPC. Test results revealed that using nPOFA instead of a higher amount of mPOFA had increased the workability of concretes, as in the case of M10N3. Thus, the introduction of nPOFA reduces the negative impact of mPOFA on concrete workability. However, it has been reported that once the content of POFA is greatly increased, more than 20%, the slump value decreases, that is, the workability of concrete decreases [21,26,49,50,51,52]. This is in good agreement with the present study where a high amount (20–30%) of mPOFA decreased the workability of concrete. This is due to the higher amount of unburnt carbon which absorbs more SP than other particles [27,53].

### 3.3. Microstructure of Concretes

**Figure 7:**
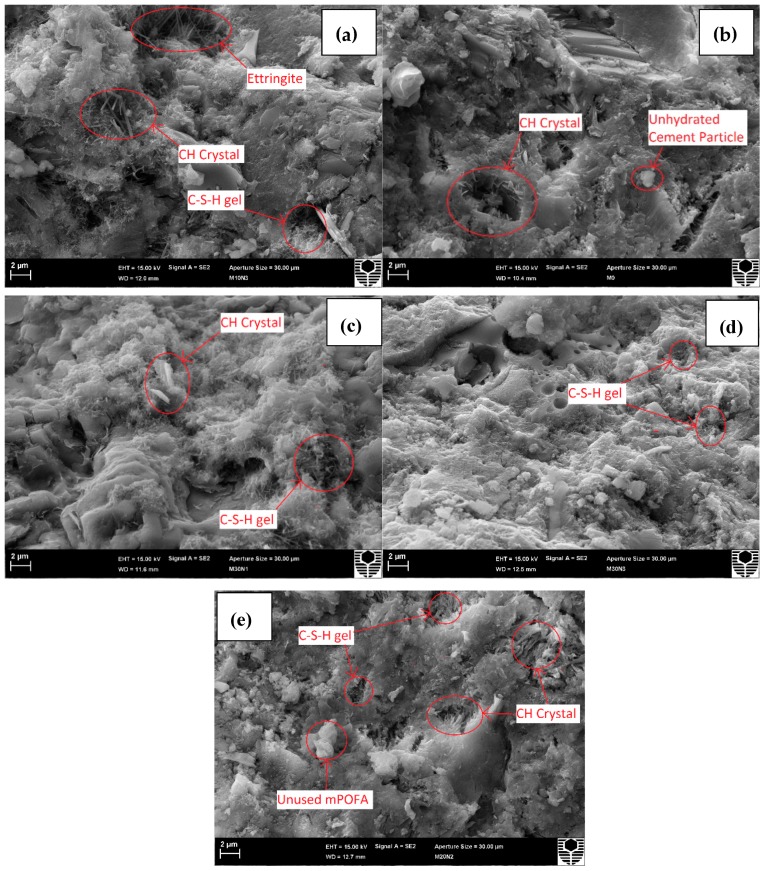
Microstructure of M0 (**a**), M10N3 (**b**), M30N1 (**c**), M30N3 (**d**) and M10N1 (**e**) after 28 days of curing.

**Figure 8:**
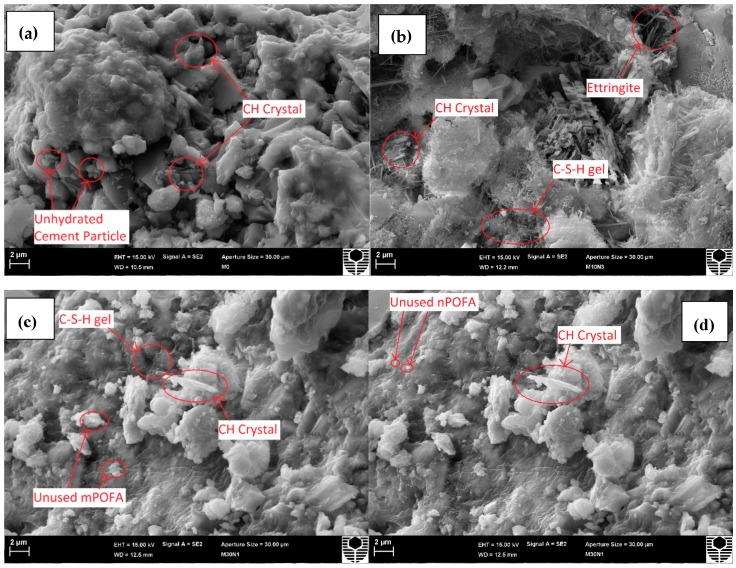
Microstructure of M0 (**a**) M10N3 (**b**) M30N1 (**c**) and M30N3 (**d**) after 28 days of curing.

There is obvious densification of concrete when incorporating mPOFA and nPOFA as shown in Figure 7. Evidently, in Figure 7a (M0, without POFA), it was hard to find C-S-H particles, while in Figure 7b–d, they are seen and marked by a circle. The other representative figures of different mixtures are shown in Figure 8. The hexagonal plate of Ca(OH)_2_ (CH, portlandite) can be seen inside the concrete pores and matrix (Figure 7a and Figure 8a) when no POFA is used. This is due to the hydration of tricalcium silicate (C_3_S) and dicalcium silicate (C_2_S) [54], which create CH after reacting with water. There is not enough SiO_2_ inside M0 (Figure 7a and Figure 8a) mixture that can consume CH to produce additional C-S-H.

POFA has been used with OPC in M10N1, M10N3, M30N1, and M30N3 concrete mixtures whose SEM images are shown in Figure 7b–e. These figures show that the aforementioned concrete mixtures had more C-S-H gel due to a higher amount of CaO and SiO_2_. The floc and fibrous C-S-H phases filled the pores of the concrete when a greater amount of POFA was incorporated, as obvious from Figure 7 and Figure 8. This concludes that POFA improves the concrete’s microstructure. Moreover, the comparison of Figure 7c (M30N1) and Figure 7d (M30N3) reveals that the microstructure of concrete including a higher amount of nPOFA had pores with smaller average size [36]. Furthermore, Rajak et al. [29] have stated that nPOFA has higher specific surface area (145.35 m^2^/g) compared with mPOFA; hence, the hypothesis given in this study correlates with their work and suggests that increased surface area induces the precipitation of hydration products [29].

In concrete mixtures, both mPOFA and nPOFA acted as nucleation sites and accelerated the precipitation of hydration products such as C-S-H gel in the cementitious matrix [16]. OPC has a high amount of calcium ions, while POFA has a high amount of silicon ions (Table 4). To balance the amount of calcium and silicon ions to develop early and late strength of concrete, it is required to use both OPC and POFA. Therefore, it is not suitable to use only OPC or a very high amount POFA with OPC, as there will not be high formation of C-S-H gel. Incorporating both OPC and POFA together at optimum proportions can produce high amount of C-S-H gel in nano sizes [18]. Higher formation of C-S-H gel can create highly compact and dense cementitious matrix. The mPOFA is small enough to fill the pores created by OPC and nPOFA can fill the pores created by mPOFA. M10N1 concrete mixture (Figure 7e) shows the highest densification in microstructure compared with other concretes. Thus, it might have the highest carbonation resistance and the lowest water sorption. Further explanations are given in Section 3.4 and Section 3.5.

The sizes of different crystals present in various concrete mixtures were calculated from Figure 7 and the results are shown in Table 6. From this table, M0 exhibits only CH crystals with 560 nm size while M30N3 does not contain CH crystals but it has C-S-H crystals with 144 nm size resulting from pozzolanic reaction. Moreover, Table 6 shows that the size of CH crystal decreases when POFA is incorporated in the concrete mixture. M30N1 and M10N1 contains 600 nm and 216 nm size CH crystals and 192 nm and 167 nm size C-S-H crystals, respectively. M10N3 contains three types of crystals such as CH, C-S-H and ettringite with 249.6 nm, 128 nm, and 219.2 nm size, respectively. The C-S-H is found to be in all 28 days old POFA concretes which fills the pores in the matrix and thus improves the quality of concrete by greater micro-filling ability.

### 3.4. Carbonation Depth

Concrete specimens were removed from the carbonation chamber and placed onto the wet cutting machine. The concrete specimens were sliced 50 mm thick after each exposure period. Phenolphthalein solution was then sprayed onto the sliced surface to identify the carbonated and uncarbonated areas. The Vernier caliper was used to measure the depth of the carbonated area.

**Figure 9:**
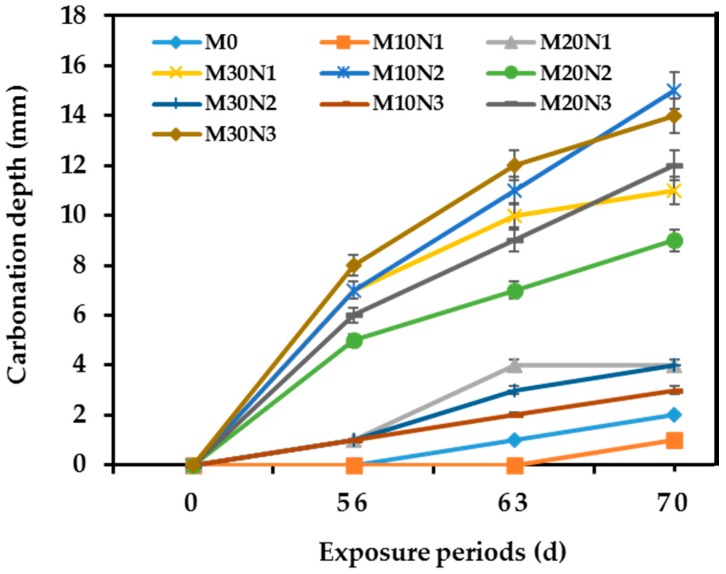
Carbonation depth of different concretes with and without POFA.

Figure 9 shows the result of carbonation depth with error bars for different mixtures in accelerated carbonation processes for 56, 63, and 70 days of exposure. The carbonation depth measurement before 56 d was not carried out because these values are too small to be seen for 5 mixtures as shown in Figure 9. However, the difference in carbonation depth can be observed after 56 d of exposure owing to the microstructural modification and chemical reaction of concrete in accelerated condition. The maximum error is found to be 5–10%. The carbonation resistance is heavily dependent on the compactness and densification of concrete, which might be influenced by the type of POFA used.

The incorporation of POFA into the concretes can influence the carbonation properties. Higher compactness and densification of concrete will lead to difficulty in penetration of CO_2_ into the concrete, thus, higher carbonation resistance can be found. However, not all mixtures have positive influence on carbonation resistance. M10N1 has the most positive effect on carbonation resistance among all concrete mixtures. Moreover, the carbonation effect tends to increase with exposure periods [27]. Therefore, the largest carbonation depth for most concretes was observed at the age of 70 days.

The presence of POFA significantly influenced the carbonation resistance of concrete. In general, the lowest and highest resistance to carbonation was observed for M30N3 (contained 30% mPOFA with 1.5% nPOFA) and M10N1 (contained 10% mPOFA with 0.5% nPOFA), respectively (Figure 9). M10N1 has higher carbonation resistance than M0 (contained 100% OPC). At 70 days of exposure, M10N1 has 1 mm of carbonation depth while M0 has 2 mm of carbonation depth. This indicates that replacing OPC with POFA increases the carbonation resistance. On the other hand, replacing too much OPC by POFA decreases the carbonation resistance. M30N3 has 14 mm of carbonation depth at the age of 70 days, which shows that it has weak carbonation resistance. POFA has a slower activity reaction compared with OPC. Hence, when a very high amount of POFA is incorporated in concrete, it requires a longer time to develop a better resistance [46]. Also, a lower amount of primary C-S-H, which contributes to pore filling, results from cement hydration when the amount of OPC is significantly reduced. It is therefore suggested that a higher amount of POFA to replace OPC is not good for carbonation resistance. The optimum amount of POFA found in this study is 10% mPOFA with 0.5% nPOFA for better carbonation resistance.

The negative impact of a relatively high amount of mPOFA on the carbonation resistance of concrete can be compensated by using nPOFA. The comparison of M10N1 with M30N1 and M10N3 shows that a higher amount of mPOFA produces lower carbonation resistance compared with higher nPOFA. When the amount of mPOFA was increased from 10% to 30%, the carbonation depth extended from 1 mm to 11 mm while increasing nPOFA from 0.5% to 1.5% increased the carbonation depth from 1 mm to only 3 mm. These results suggested that nano-sized POFA has a better ability to resist the penetration of CO_2_ into concrete than micro-sized POFA. Nano-sized POFA has a better micro-filling ability to reduce porosity causing difficulties to penetrate CO_2_ into concrete. Therefore, using nPOFA provided higher carbonation resistance than mPOFA.

In summary, the higher amount of POFA incorporated in concrete increases the number of pores with greater porosity, resulting in a higher carbonation depth (lower carbonation resistance). This is because POFA had a slower pozzolanic reaction [36] and the production of C-S-H is reduced. Therefore, there was higher chance of increasing the carbonation resistance by increasing the duration of curing days.

### 3.5. Water Sorption

**Figure 10:**
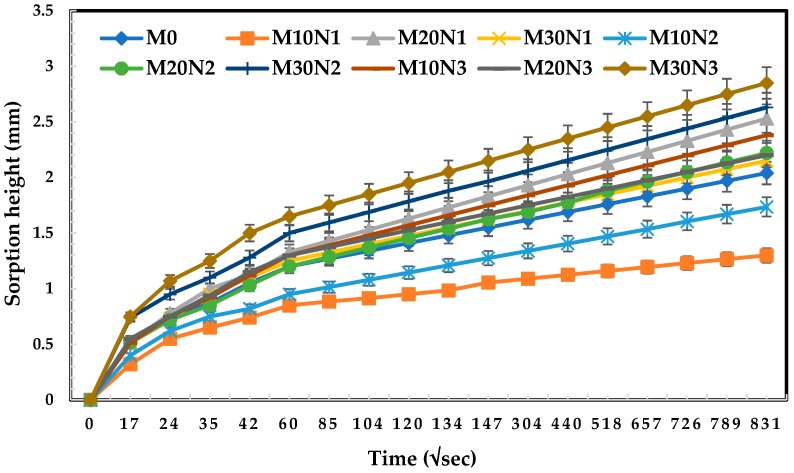
Water sorption of different concretes with and without POFA.

The results of sorptivity test with error bars for different 28 days old concrete mixtures with various proportions of OPC and POFA are shown in Figure 10. This figure shows that M30N3 (contained 30% mPOFA and 1.5% nPOFA) had the largest water sorption while M10N1 (contained 10% mPOFA and 0.5% nPOFA) had the smallest water sorption. The highest water sorption of M30N3 is attributed to the largest porosity present in the concrete which correlates with the SEM images (Figure 7 and Figure 8) and carbonation depth measurement (Figure 9). However, M10N1 had the lowest water sorption, which suggests that it had the highest penetration resistance and therefore it provided the lowest carbonation depth.

The water sorptivity values increased with time for all concretes due to the suction mechanism of capillary water molecules. The incorporation of a certain amount of POFA (10%) into concrete showed a greater decrease in sorptivity, which means that it provided a higher resistance to moisture absorption by capillary suction. This indicates that more binding product (C-S-H) formed to refine the pore structure in the concrete matrix, thus producing a denser microstructure in the concrete.

The lowest levels of water sorptivity were observed for M10N1 and M10N2, both of which contained 10% mPOFA but 0.5% and 1% nPOFA, respectively. The time for suction of the water molecules through the pores of concrete was lower for M10N1 compared with other concrete mixtures, thus less water sorptivity was observed. Interestingly, although M10N2 had relatively high carbonation depth, it provided significantly low water sorption value, as compared to other concretes except M10N1. This perhaps occurred because the water sorptivity depends not only on the microstructure and compactness of concrete but also on the different processes of experiment. The sorptivity experiment was performed after 28 days of water curing and then 1 min–60 min alternation in wetting time. There is a possibility that after curing in water, when the sorptivity experiment was performed, the water absorption was less due to the reduced degree of dryness in concrete. In contrast, the carbonation measurement was carried out for up to 70 days where the likelihood for dryness was greater, which caused the formation of greater large-size pores in the concrete. Therefore, it showed less carbonation resistance.

## 4. Conclusions

Based on the experimental results obtained in the present study and associated discussion, the following conclusions can be drawn:

(a) XRF results show that POFA has higher SiO_2_ as well as SiO_2_ + Al_2_O_3_ + Fe_2_O_3_ compared with OPC.
(b) The LOI value of treated POFA is greatly reduced compared with raw POFA. After the treatment of raw POFA, the LOI value is reduced by 28.75% and 33.33% for mPOFA and nPOFA, respectively.
(c) XRD results reveal that mPOFA and nPOFA contain two major phases, namely Quartz and Cristobalite, along with amorphous silica, which participated in pozzolanic reaction.
(d) SEM results show that mPOFA had crushed or irregular-shaped particles and found to be bigger in size compared with nPOFA.
(e) Inclusion of nPOFA can reduce the size of pores in the concrete matrix due to its better micro-filling ability than mPOFA.
(f) The concrete mixture with 10% mPOFA and 0.5% nPOFA, designated as M10N1, can have higher carbonation resistance and lesser sorptivity compared with OPC and other mixtures. However, a higher amount of micro- and nano-POFA has detrimental and negative effects on carbonation resistance and water sorptivity results.

## Figures and Tables

**Table 1:** Binder composition of different concretes.

Mixture	OPC	mPOFA	nPOFA	Mixture	OPC	mPOFA	nPOFA
M0	100%	0%	0%	M20N2	79.0%	20%	1.0%
M10N1	89.5%	10%	0.5%	M30N2	69.0%	30%	1.0%
M20N1	79.5%	20%	0.5%	M10N3	88.5%	10%	1.5%
M30N1	69.5%	30%	0.5%	M20N3	78.5%	20%	1.5%
M10N2	89.0%	10%	1.0%	M30N3	68.5%	30%	1.5%

**Table 2:** Batch weights (kg) of different constituent materials in various concretes (Batch volume = 0.001373 m^3^)

Mixture	Cement	POFA		Coarse Aggregates	Quarry Dust	Sand		Water	SP Dosage (0.2% of Binder *)
		Micro	Nano			Wet	Dry		
**M0**	2.9	0	0	5.40	1.33	0.266	1.06	0.90	0.0058
**M10N1**	2.6	0.288	0.0144	5.40	1.33	0.266	1.06	0.90	0.0058
**M20N1**	2.31	0.58	0.0144	5.40	1.33	0.266	1.06	0.90	0.0058
**M30N1**	2.02	0.871	0.0144	5.40	1.33	0.266	1.06	0.90	0.0058
**M10N2**	2.58	0.288	0.0288	5.40	1.33	0.266	1.06	0.90	0.0058
**M20N2**	2.3	0.58	0.0288	5.40	1.33	0.266	1.06	0.90	0.0058
**M30N2**	2	0.871	0.0288	5.40	1.33	0.266	1.06	0.90	0.0058
**M10N3**	2.57	0.288	0.0432	5.40	1.33	0.266	1.06	0.90	0.0058
**M20N3**	2.28	0.58	0.0432	5.40	1.33	0.266	1.06	0.90	0.0058
**M30N3**	1.99	0.871	0.0432	5.40	1.33	0.266	1.06	0.90	0.0058

* Binder: Cement plus POFA.

**Table 3:** Sorptivity time intervals used in present study according to ASTM C1585-13 [44].

**Time**	1 min	5 min	10 min	20 min	30 min	60 min
**Tolerance**	2 s	10 s	2 min	2 min	2 min	2 min

**Table 4:** Chemical compositions of OPC and POFA.

Chemical Composition (%)	OPC	Raw POFA	mPOFA	nPOFA
SiO_2_	16.40	59.1	69.19	68.07
Al_2_O_3_	4.24	4.5	3.34	3.71
Fe_2_O_3_	3.53	6.5	3.19	3.24
CaO	68.30	8.6	6.70	7.41
MgO	2.39	2.6	4.65	5.10
SO_3_	4.39	2.7	0.605	0.626
SiO_2_ + Al_2_O_3_ + Fe_2_O_3_	24.17	70.1	75.19	75.02
LOI	2.40	10.5	1.71	1.60

**Table 5:** Slump test results.

Mixture	Slump Value (mm)	Slump Loss (mm)	Mixture	Slump Value (mm)	Slump Loss (mm)
M0	140	0	M20N2	65	75
M10N1	125	15	M30N2	45	95
M20N1	50	90	M10N3	145	−5
M30N1	40	100	M20N3	80	60
M10N2	140	0	M30N3	65	75

**Table 6:** Average size (nm) of crystals present in different mixtures.

Mixture	Crystal Size (nm)		
	CH	C-S-H	Ettringite
M0	560	-	-
M10N3	249.6	128	219.2
M30N1	600	192	-
M30N3	-	144	-
M10N1	216	167	-
